# Desensitization to Tocilizumab in a Patient With Juvenile Idiopathic Arthritis

**DOI:** 10.7759/cureus.81084

**Published:** 2025-03-24

**Authors:** Rosalaura V Villarreal-González, Diana E Cadenas-García, Adriana Vázquez-Nungaray, Ana V Villarreal-Treviño, Oscar Vidal-Gutiérrez

**Affiliations:** 1 Oncology, University Hospital “Dr. José Eleuterio González”, Faculty of Medicine, Autonomous University of Nuevo León, Monterrey, MEX; 2 Allergy and Immunology, Instituto de Seguridad y Servicios Sociales de los Trabajadores del Estado (ISSSTE) Hospital “Dr. Valentín Gómez Farías”, Zapopan, MEX; 3 Pediatric Rheumatology, University Hospital “Dr. José Eleuterio González”, Faculty of Medicine, Autonomous University of Nuevo León, Monterrey, MEX; 4 Oncology, Universidad Autónoma de Nuevo León, Monterrey, MEX

**Keywords:** allergy and anaphylaxis, desensitization therapy, drug hypersensitivity reaction, juvenile idiopathic arthritis, tocilizumab

## Abstract

Juvenile idiopathic arthritis is the most common chronic inflammatory rheumatic disease in children, classified by the number of affected joints, systemic symptoms, and rheumatoid factor presence. We describe the case of an eight-year-old female with extended oligoarticular juvenile idiopathic arthritis and a strong family history of atopy who developed a Grade II hypersensitivity reaction during a tocilizumab infusion after four years of treatment. Symptoms, including rash, urticaria on the neck and arms, periauricular edema, tachycardia, and hypertension, emerged within 30 minutes of infusion. The reaction was managed with intramuscular epinephrine, intravenous hydrocortisone, and saline, leading to symptom resolution. Skin prick testing was negative, but intradermal testing showed a positive reaction. Due to the drug’s effectiveness in controlling her disease, a desensitization protocol was initiated, involving a 12-step, 5.67-hour infusion schedule with premedication every four weeks. This case highlights the importance of desensitization protocols in patients requiring continued monoclonal antibody therapy despite hypersensitivity reactions.

## Introduction

Juvenile idiopathic arthritis (JIA) is the most common chronic inflammatory rheumatic condition of childhood, with a global incidence of 1.6 to 23 per 100,000 and a prevalence of 3.8 to 400 per 100,000 [1]. The International League of Associations of Rheumatology classifies subtypes based on the number of affected joints, systemic symptoms, and the presence of rheumatoid factor [2]. Treatment of JIA is categorized into non-biological treatments (anti-inflammatory drugs, corticosteroids, and disease-modifying antirheumatic drugs such as methotrexate, sulfasalazine, and leflunomide) and biological therapies, which target specific proinflammatory cytokines involved in JIA pathogenesis, such as interleukin (IL)-1, IL-6, and tumor necrosis factor [3].

Tocilizumab is a humanized monoclonal antibody targeting IL-6, primarily used in the treatment of systemic and polyarticular JIA. IL-6 plays a critical role in the pathogenesis of JIA by driving inflammation, joint destruction, and systemic manifestations, making it a key target for monoclonal antibody therapy. Hypersensitivity reactions to this monoclonal antibody are estimated to occur in 0.1% to 0.7% of pediatric cases in previous studies [4].

The pathogenesis of hypersensitivity reactions is known to involve cytokine or IgE-mediated release reactions, which include mediators such as histamine, leukotrienes, prostaglandins, tryptase, and platelet-activating factors. Systemic clinical manifestations include urticaria, angioedema, flushing, cough, dyspnea, nausea, vomiting, and hypotension. IgE-mediated anaphylaxis is characterized as a severe, life-threatening systemic hypersensitivity reaction. Additionally, delayed drug reactions with eosinophilia and systemic symptoms have been reported in patients receiving tocilizumab in a few cases [5,6].

## Case presentation

**Figure 1:**
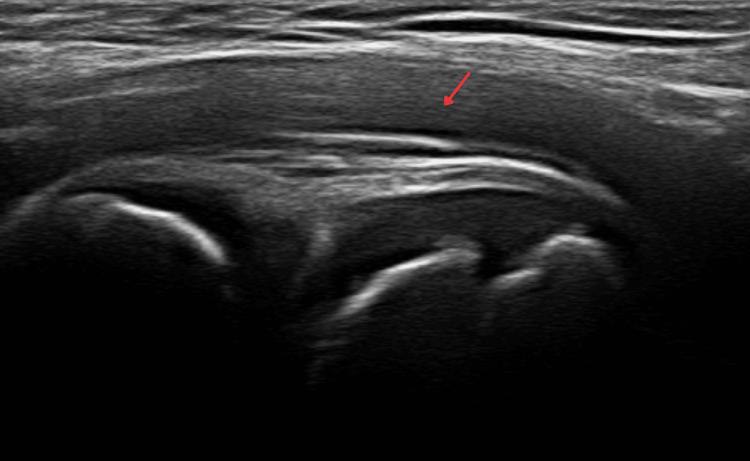
Ultrasound of the right hip joint at the time of juvenile idiopathic arthritis diagnosis. Bilateral hip joint effusion, more prominent on the right side. No Doppler power signal was detected (exudative synovitis). The remainder of the study was within normal parameters.

We describe the case of an eight-year-old female diagnosed with extended oligoarticular JIA (Figure 1) with a family history of atopy, including allergic rhinitis, atopic dermatitis, urticaria, drug allergy, food allergy, allergy to Hymenoptera sting, and anaphylaxis.

During a tocilizumab infusion, 30 minutes into the procedure after four years of treatment, the patient developed a hypersensitivity reaction, presenting with a rash, urticaria on the neck and arms, periauricular edema, tachycardia at 115 beats/minute, and hypertension at 90/50 mmHg (with a baseline of 60/30 mmHg). The hypersensitivity reaction was classified as Grade II on Brown’s scale (Table 1). Epinephrine 0.50 mg was administered intramuscularly, along with 250 mL of physiological saline and 30 mg of hydrocortisone intravenously, resulting in the resolution of symptoms.

**Table 1:** Brown’s scale. The table is adapted from Picard and Galvão [7].

Grade	Severity	Description
1	Mild	Symptoms are limited to the skin or involve a single organ/system and are mild
2	Moderate	Symptoms involve at least two organs/systems, but there is no significant drop in blood pressure or oxygen saturation
3	Severe	Symptoms typically involve at least two organs/systems and there is a significant drop in blood pressure (systolic ≤90 mmHg and/or syncope) and/or oxygen saturation (≤92%)

Two weeks later, a skin prick test of tocilizumab with a concentration of 20 mg/mL was performed with a negative result. Intradermal skin tests at 0.2 mg/mL were positive (8 × 8 mm) compared to the negative control (glycerinated solution 2 × 2 mm).

**Figure 2:**
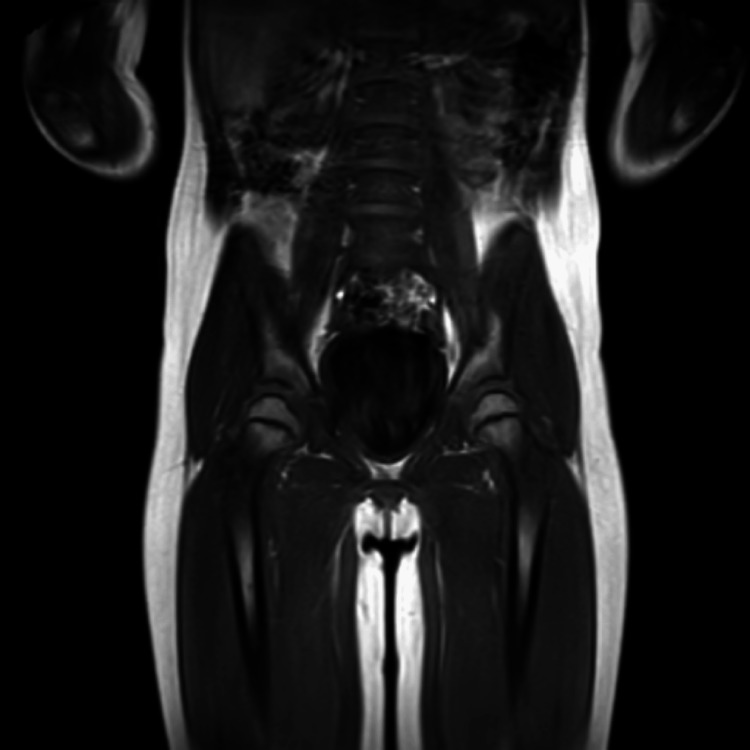
MRI study (T1) after five cycles of tocilizumab desensitization, with the disease in a non-active state. The lower dorsal region, lumbar spine, sacrum, and coccyx show no abnormalities in vertebral bodies or intervertebral spaces. The spinal cord and cauda equina appear normal. The sacroiliac and hip joints show no alterations and no evidence of edema or joint effusion. The thigh muscles up to the mid-third of the femur show no abnormalities, and the signal intensity of the femur up to the mid-third is normal.

Due to the adequate response of the disease to the monoclonal antibody, it was decided to initiate a desensitization protocol to tocilizumab 240 mg, consisting of three bags and 12 steps over 5.67 hours (Tables 2, 3). Premedication with levocetirizine 5 mg orally was administered one hour before the infusion. The procedure was completed successfully without any hypersensitivity reactions, repeated every four weeks (Figure 2).

**Table 2:** Preparation of the medication for the tocilizumab desensitization protocol in three bags.

Three bags	Volume (mL) per bag	Concentration (mg/mL) per bag	Amount (mL) of bag infused	Infused dose (mg) per bag
Solution 1	50	0.024 mg/mL	3.75	0.09 mg
Solution 2	50	0.24 mg/mL	7.5	1.8 mg
Solution 3	100	2.38 mg/mL	100	238.11 mg

**Table 3:** Three-bag, 12-step desensitization protocol to tocilizumab.

Step	Solution	Rate (mL/hour)	Time (minute)	Volume infused (mL)	Administered dose (mg)	Cumulative dose (mg)
1	1	1	15	0.25	0.006 mg	0.006 mg
2	1	2	15	0.5	0.012 mg	0.018 mg
3	1	4	15	1	0.024 mg	0.042 mg
4	1	8	15	2	0.048 mg	0.09 mg
5	2	2	15	0.5	0.12 mg	0.21 mg
6	2	4	15	1	0.24 mg	0.45 mg
7	2	8	15	2	0.48 mg	0.93 mg
8	2	16	15	4	0.96 mg	1.89 mg
9	3	4	15	1	2.38 mg	4.27 mg
10	3	8	15	2	4.76 mg	9.03 mg
11	3	16	15	4	9.52 mg	18.55 mg
12	3	32	15	93	221.45 mg	240 mg
Time: 5.67 h						Total dose: 240 mg

## Discussion

JIA is classified into oligoarticular (persistent or extended), polyarticular (rheumatoid factor positive or negative), systemic, psoriatic arthritis, and enthesitis-related arthritis. The type of JIA influences genetic susceptibility and disease severity [3].

We describe a pediatric patient with extended oligoarticular JIA who developed a hypersensitivity reaction to tocilizumab and underwent a desensitization protocol. Hypersensitivity reactions to monoclonals can be infusion-related reactions, cytokine release reactions, type I reactions (which can be IgE-mediated or non-IgE-mediated), type III reactions, and late type IV reactions. Infusion-related reactions and cytokine release reactions to monoclonal antibodies can occur from the first infusion and may present with moderate to severe symptoms [8].

In a recent study of patients with JIA treated with tocilizumab, hypersensitivity reactions were reported to occur within 35 minutes to five hours after administration, with symptoms including fever, respiratory symptoms (cough, desaturation), gastrointestinal symptoms (vomiting, abdominal pain), and cardiovascular symptoms (tachycardia, bradycardia, hypotension). Risk factors for hypersensitivity reactions to tocilizumab included younger age and higher disease activity [9].

The first report of rapid desensitization of a pediatric patient in a Latin American population was of eight steps, with a dilution of tocilizumab (0.47 mg/mL in 250 mL of 0.9% physiological solution), successfully performed [10]. There are few reported cases in the literature of desensitization to tocilizumab in pediatric patients. One such case involved a 15-year-old boy with JIA who presented with Grade 2 anaphylaxis after the second dose of tocilizumab. The desensitization protocol consisted of 12 steps with three bags of tocilizumab (600 mg), and lasted five hours and 45 minutes, with no adverse events [11].

Currently, monoclonal antibodies constitute a cornerstone in the treatment of numerous diseases; therefore, an increasing number of adverse reactions are being documented, underscoring the importance of desensitization protocols.

## Conclusions

This case underscores the challenges of managing hypersensitivity reactions in patients undergoing biologic therapy. Despite four years of effective treatment with tocilizumab for extended oligoarticular JIA, the patient developed a Grade II hypersensitivity reaction, which was promptly resolved with epinephrine, hydrocortisone, and physiological saline. Therefore, the implementation of a desensitization protocol was required. The successful desensitization process enabled the continuation of treatment without further hypersensitivity episodes, highlighting the importance of such protocols for patients reliant on biologic therapies. With the increasing use of monoclonal antibodies in the treatment of various diseases, this case emphasizes the necessity of proactive measures and individualized strategies to manage hypersensitivity reactions while ensuring patient safety and therapeutic efficacy.
